# Cecum microbiota composition, fermentation characteristics, and immunometabolic biomarkers of Yunshang black goat fed varying dietary energy and protein levels

**DOI:** 10.3389/fmicb.2025.1523586

**Published:** 2025-02-04

**Authors:** Binlong Fu, Xiaoqi Zhao, Muhammad Khan, Yanting Jiang, Weijuan Li, Maida Mushtaq, Baiji Danzeng, Xiaojun Ni, Zobia Azeem, Qingyong Shao, Bai Xue, Yina Ouyang

**Affiliations:** ^1^Yunnan Animal Sciences and Veterinary Institute, Kunming, China; ^2^Department of Zoology, University of Veterinary and Animal Sciences, Lahore, Pakistan; ^3^Animal Nutrition Institute, Sichuan Agricultural University, Chengdu, China

**Keywords:** Yunshang black goat, dietary energy and protein, cecal microbiota composition, fermentation characteristics, immunometabolic biomarkers

## Abstract

**Introduction:**

Ruminants including goats have diverse microcosms of microbiota involved in diet digestion, absorption, and assimilation. Moreover, it is well known that changes in dietary regimens including nutrient levels result in varied gut microbiota composition, and ultimately, the performance and health of these animals.

**Methods:**

The current study examined the effects of varying dietary energy and protein levels on the cecal fermentation, immune biomarkers, and microbiota characteristics of 80 male Yunshan Black Goats (6 months, ~35.82 ± 2.79 kg), divided into four diets: 1) High Energy-High Protein (HEHP), 2) High Energy-Low Protein (HELP), 3) Low Energy-High Protein (LEHP), and 4) Low Energy-Low Protein (LELP). Twenty goats (five from each treatment group) were randomly slaughtered after a 50-day feeding trial, and cecal digesta and tissue were sampled for microbial analysis.

**Results:**

The cecal content revealed that the high-energy groups (HEHP, HELP) had lower pH levels than the LEHP group (*p* < 0.05) and significantly higher valeric and isovaleric acid concentrations in HEHP. Although species richness (Chao1 index) remained consistent, the HEHP group showed higher diversity (Shannon and Simpson indices) than LEHP (*p* < 0.05). Dominant phyla included *Bacteroidetes* and *Firmicutes*; LEHP and LELP had significantly higher *Bacteroidetes* abundance than HELP, while HELP had higher *Firmicutes* abundance than LEHP (*p* < 0.05). *Verrucomicrobia* abundance was lower in LEHP than in HELP and LELP (*p* < 0.05). At the genus level, 311 genera were identified, with *Clostridium*, *Prevotella*, *unidentified_BS11*, and others showing significant variation. The HELP group had lower *unidentified_BS11* than LEHP and LELP, and higher *unidentified_Ruminococcaceae*, *Clostridium*, and *Lachnospiraceae* than LEHP (*p* < 0.05). VFA metabolism, absorption, cytokine expression, and tight junction protein mRNA in cecal tissue were also analyzed. Genes like MCT-1 and SLC16A4, linked to VFA absorption, positively correlated with *Paludibacter*, which was associated with immune markers (TLR-3, TLR-4, IFN-*γ*) and Occludin expression. In contrast, VFA-related genes and tight junction proteins negatively correlated with *unidentified Fibrobacterales*, suggesting a microbial role in adaptive immunity.

**Conclusion:**

This study demonstrated that dietary energy and protein levels significantly influenced cecal fermentation, immune biomarkers, and microbiota composition in Yunshan Black Goats.

## 1 Introduction

Ruminants including goats have multi-comparted stomach, which host a diverse and ecologically complex microbiome that is crucial to their digestive efficiency, health, and performance. In the rumen, this microbiome ferments a variety of carbohydrates sugars, starch, cellulose, hemicellulose, and pectin yielding carbon dioxide, methane, hydrogen, and volatile fatty acids (VFAs) (Salgado-Flores et al., 2016). However, accumulating evidence highlights the significant role of the hindgut and its microbial residents in maintaining ruminant health and productive efficiency (Gressley et al., 2011). Understanding the interactions between host and microbiome in the hindgut, particularly in the cecum, is critical as these relationships impact nutrient utilization, animal health, and reproductive performance (Gressley et al., 2011).

The cecum, a major fermentation site within the ruminant hindgut, is vital for nutrients digestion and intestinal health. Upon entry into the hindgut, undigested fiber components, such as cellulose and hemicellulose, undergo secondary fermentation by the cecal microbiome, producing VFAs that supply additional energy to the host and enhance feed utilization efficiency (Gressley et al., 2011). Additionally, the cecum contributes significantly to overall animal health, productivity, and welfare (Salgado-Flores et al., 2016). Unlike the rumen, the cecum lacks natural buffering mechanisms like protozoa and saliva, possesses a distinct epithelial structure, and has a limited capacity to neutralize low pH (Gressley et al., 2011).

The metagenomic studies have revealed that high-grain diets alter the composition and metabolism of the cecal microbiome and can induce mucosal damage (Slyter, 1976). The effects of nutritional imbalances on the cecal microbiome, epithelial metabolism, and immune responses in pregnant sheep are well established (Xie et al., 2021). However, the interactions between the cecal microbiome and fatty acid production, and the microbiome’s response to dietary changes in protein and energy levels, remain poorly understood (Wu et al., 2023). Dietary protein levels that exceed the small intestine’s absorptive capacity may increase bacterial protein fermentation in the hindgut, producing inflammatory metabolites linked to hindgut acidosis (Liu et al., 2014). Therefore, the current study hypothesized to investigate how changes in dietary energy and protein affect the composition and function of the cecal microbiome and their implications for gene expression in cecal epithelial tissues. Through quantitative PCR, we measure the expression of genes involved in VFA absorption and metabolism, cytokine production, and tight junction proteins. The current study aimed to deepen understanding of host-microbiome interactions in response to dietary shifts and offer valuable insights for optimizing the nutritional management of Yunshang black goats.

## 2 Materials and methods

### 2.1 Experimental site, animals, and study design

The current experiment was conducted at the Yunnan Academy of Animal Husbandry and Veterinary Sciences research facility, after approval from the ethical committee of the Yunnan Academy of Animal Husbandry and Veterinary Sciences (201911004). In this study, 80 male Yunshang Black Goats (approx. 6 months old, 35.82 ± 2.79 kg) were assigned to four diets in a completely randomized 2 × 2 factorial design: (1) high energy-high protein (HEHP), (2) high energy-low protein (HELP), (3) low energy-high protein (LEHP), and (4) low energy-low protein (LELP). The high-energy diets provided 9.74–9.76 MJ/kg, and high-protein diets contained 12.99–13.04% protein, while low-energy diets provided 8.14–8.18 MJ/kg, and low-protein diets contained 10.01–10.05% protein. After a 14-day adaptation, a 50-day feeding trial was conducted. Goats, housed individually in 1.5 × 2.0 m pens, had *ad libitum* feed and water access, with feed offered twice daily at 08:00 and 17:00, adjusting by 5% daily to ensure sufficient intake.

### 2.2 Slaughtering and sample collection

After the feeding trial, twenty goats (five from each treatment group) were randomly chosen, and were transported to the slaughtering facility early in the morning after a 24 h feed and water withdrawal. The goats were then electrically stunned and slaughtered following animal welfare guidelines. The digestive tract was dissected, and the cecum was isolated immediately. Cecal digesta samples were collected, with pH measured on-site using a pH meter (Testo-205, Germany). Samples were diluted with distilled water, centrifuged at 2000 g for 10 min, and the supernatant mixed with 25% (wt/vol) metaphosphoric acid (5 mL supernatant and 1 mL acid) before storage at -20°C for VFAs analysis via gas chromatography (GC-14B; Shimadzu, Japan). Cecal epithelium samples were washed with ice-cold phosphate-buffered saline and divided. One portion, cut into 0.4 × 0.4 cm sections, was stored at -80°C for RNA extraction, while the other was fixed in 4% paraformaldehyde for histomorphometric microscopy. Briefly, about 7 cm of cecum, was washed the chime on each tissue block with normal saline, and then fixed in 4% formaldehyde solution. The cecal sections were made by paraffin and hematoxylin–eosin (HE) staining, and the morphology of the cecal epithelium was observed by panoramic section scanner (3DHISTECH, PANNORAMIC, Hungary).

### 2.3 Cecal fermentation analysis

Cecal digesta VFAs were analyzed by gas chromatography (GC-14B; Shimadzu, Japan). Briefly, samples were diluted 1:5 with distilled water, centrifuged at 2000 g for 10 min, and the supernatant was mixed with 25% (w/v) metaphosphoric acid to assess VFA concentrations. The gas chromatograph, fitted with a 30 m × 0.32 mm × 0.25 μm capillary column, operated at a column temperature of 130°C, vaporization temperature of 180°C, and a flame ionization detector at 180°C. VFA molar proportions were calculated by dividing individual VFA concentrations by the total VFA concentration.

### 2.4 16S rRNA gene sequencing analysis of cecal digesta

Microbial RNA from cecal digesta was extracted using the cetyltrimethylammonium bromide (CTAB) method. The V3–V4 region of the 16S rRNA gene was amplified with universal primers 341F and 806R. Amplicons were purified with the AxyPrep DNA Gel Extraction Kit (Axygen Biosciences, United States), and sequencing libraries were prepared using the Illumina TruSeq DNA Sample Preparation Kit. Cluster generation, template hybridization, and amplification were performed with the TruSeq PE Cluster Kit and TruSeq SBS Kit, and sequencing was conducted on an Illumina MiSeq platform. Raw data were processed in QIIME (v1.9.0), with operational taxonomic units (OTUs) clustered at 97% similarity using UPARSE (v7.1) and chimeras removed via UCHIME. Taxonomic analysis of OTUs representatives was completed using the RDP Classifier (v2.2) with reference to the SILVA database (v132).

### 2.5 RNA extraction, reverse transcription, and qPCR analysis

All digesta and mucosa samples were thawed at 4°C. Approximately 200 mg of each sample was placed into 2 mL centrifuge tubes containing 0.2 g of bead powder. By the provided instructions, total RNA was extracted by employing the RNAiso Plus Total RNA Extraction Kit (TaKaRa, Dalian, China), and its integrity was examined via 1.5% agarose gel electrophoresis (Biowest Agarose, Spain). The concentration and OD value were determined using the NanoDrop 2000 UV–Visible Spectrophotometer (Thermo Fisher Scientific, Waltham, MA, United States). Eventually, cDNA was synthesized with the utilization of Oligo (dT)_18_ primer (50 pmol/μL) and M-MLV reverse transcriptase (TaKaRa, Dalian, China) following the instructions (Table 1). The three internal reference genes, namely ACTB (entry number: NM_001290932), GAPDH (entry number: XM_006065800), and RPS23 (entry number: XM_006059350), were employed as controls in this study. Each PCR Strip & Caps tube was supplemented with 10 μL of dib^®^ SYBR qPCR SuperMix Plus (Data Invention Biotech, ABC Biochemistry, China). Additionally, 0.8 μL of upstream and downstream primers (1 μM), 2 μL of template cDNA (100 ng/μL), and 6.4 μL of ddH_2_O were prepared using the iQ^5^ Real-Time PCR system (Bio-Rad Laboratories, United States) for RT-qPCR analysis with three replicates for all data. The experimental procedure involved predenaturation at 95°C for 30 s, denaturation at 95°C for 5 s, annealing at 60°C for 30 s, and extension at 72°C for 30 s over a total of 40 cycles. Finally, the calculation method of 2^−△△Ct^ was used for relative quantitative analysis. Relative quantitative analysis was conducted using the ΔΔCt method, where ΔCt = Ct_(target gene)_ − Ct_(geometric mean of internal reference gene)_ and ΔΔCt = ΔCt − ΔCt_(median)_.

**Table 1:** Primer sequence used for qPCR analysis.

Genes	Primer (5′ → 3′)	Source	Size (bp)	Temperature (°C)
Claudin-1F	TGCCCCAGTGGAAGGTTTAC	XM_005675123.3	266	60
Claudin-1R	CTTCTGTGCCTCGTCGTCTT			
Claudin-4 F	CGTGCTATTGTCGGTGGTGG	XM_005697785.2	300	60
Claudin-4 R	GAGTAGGGCTTGTTGTTTCGTG			
Occludin F	GCGGTAACTTGGAGACGCTTTC	XM_018065677.1	107	60
Occludin R	GCCTCCCGTCGTGTAGTCTGTT			
ZO-1 F	TTGAACGCAAGTTTGAAAGTCCT	XM_018066118.1	121	60
ZO-1 R	TGTGCAGACTTCAGGAGGGTTT			
BDH-1 F	CCAGGACTACGGCAGGAAGTA	XM_018046459.1	143	60
BDH-1 R	GGTGGTAGCGAGTGTAGGGAGT			
BDH-2 F	AAGGCGTTGTGAACAGGTGC	XM_005681356.3	182	60
BDH-2 R	CAGTGCCTCTTCAGGATTTGGT			
HMGCL F	CTAAAGTCGCTGAGGTCTCCAAG	XM_005676858.2	225	60
HMGCL R	AGTCCACGACACTCACTCCCAT			
HMGCS1 F	CGATGGTGTAGACGCTGGAAAG	XM_005694739.3	150	60
HMGCS1 R	CGCCCAATGCAGTCATAAGAAA			
DRA F	AATTGTGGGCTATTAAAGAGGACC	NM_001314188.1	230	60
DRA R	CATGATGTCCAGGTTGGCTTTC			
NHE1F	CTGGACATTTGTTATCAGCACCCT	XM_018056627.1	177	60
NHE1R	AGGAGGTAGCCCAGGGAGAA			
SLC9A2 F	TTCGGGAGAACCAACCCAAG	XM_018054964.1	224	60
SLC9A2 R	CAAGGTTCGCTGACGGATCT			
SLC9A3 F	CCCGGCAGGAGTACAAACAT	XM_018065648.1	125	60
SLC9A3 R	CTTGGCCGACTTGAAGGACT			
MCT1F	CCCTCAACCAGGCTTTCTTTAT	XM_013962525.2	116	60
MCT1R	CTTTGGCCCTATTGGTCTCATC			
SLC16A4 F	GGAGCTATCACATTGCATCTGGTT	XM_013962551.2	115	60
SLC16A4 R	CTGGACCACTTGGAGACAAACTACT			
TLR3 F	ATCTGTCCCTGAGCAGCAACC	XM_018041934.1	131	60
TLR3 R	GGCAAAGGAGTCATTACCCATC			
TLR4 F	CTTCCCAACCTGGAGCACTT	NM_001285574.1	128	60
TLR4 R	TCTAAAGGGTTCAGGGACAAATCTA			
IFN-γF	TGGAAAGAGGAGAGTGACAAAAAG	NM_001285682.1	101	60
IFN-γR	CTCCTTTGAATGACCTGGTTATCT			
IL-6 F	TGAAGGAAAAGATCGCAGGTCTAA	NM_001285640.1	100	60
IL-6 R	ACCTTTGCGTTCTTTACCCACT			

### 2.6 Bioinformatics and statistical analysis

The Shapiro–Wilk and Bartlett tests were performed using R software version 3.5.1 (R Core Team, Vienna, Austria) to evaluate the normality and homogeneity of variance across all data distributions. Initial data processing was conducted in Excel 2016, followed by analysis using SPSS v24 statistical software. For the *α* diversity index, Student’s *t*-test was applied. Additionally, a one-way analysis of variance (ANOVA) was conducted, with the least significant difference (LSD) test employed for multiple comparisons. Spearman’s correlation coefficient was calculated to assess correlations between microbial species and intestinal metabolites. Data of qPCR were analyzed using GraphPad Prism (Version 9.0.0, San Diego, California, United States) for visualization purposes, and an independent sample T-test was utilized to determine statistical significance (*p* < 0.05).

## 3 Results

### 3.1 Cecal fermentation characteristic

**Figure 1:**
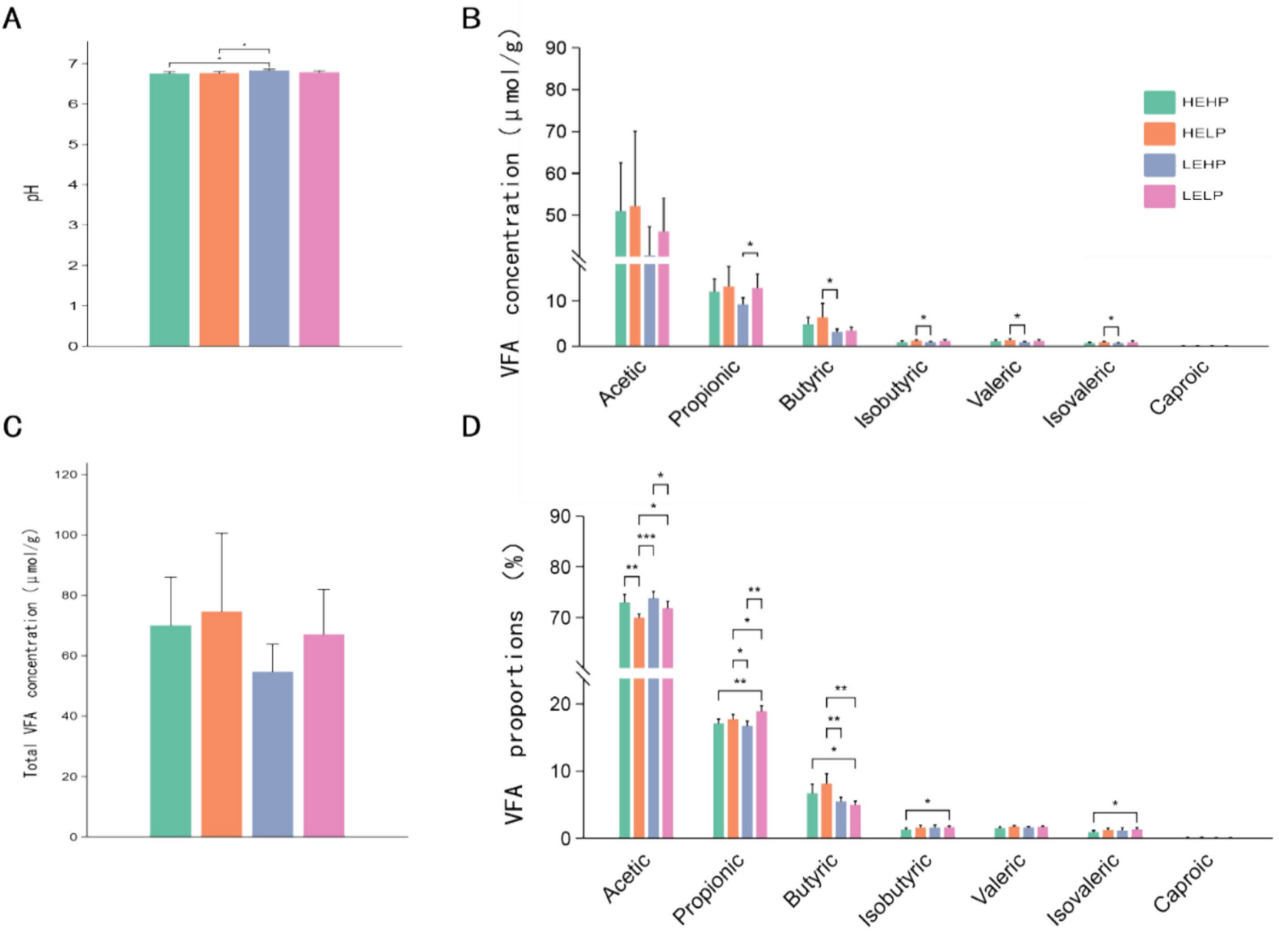
The effect of dietary protein and energy levels on pH **(A)**, relative concentration **(B)**, total VFA content **(C)**, and proportions of acetic acid, propionic acid, butyric acid, isobutyric acid, valeric acid, and caproic acid **(D)** in goat cecal digesta. Significant differences between treatments were determined using the Kruskal-Wallis test. *, **, and ***represent *p* < 0.05, *p* < 0.01, and *p* < 0.001, respectively.

The dietary protein and energy levels have influenced (*p* < 0.05) the cecal fermentation of the goats (Figure 1). The pH levels in the HEHP and HELP groups were notably lower compared to the LEHP group (*p* < 0.05), while no significant differences were observed between the LELP group and the others (*p* > 0.05) (Figure 1A). The propionate concentration in the LEHP group was significantly lower than in the LELP group (*p* < 0.05). Additionally, the HEHP group exhibited significantly higher concentrations of butyric, isobutyric, valeric, and isovaleric acids compared to the LEHP group (*p* < 0.05) (Figure 1B). Acetate was the predominant VFA across all groups, comprising approximately 70.0% of the total VFAs, followed by propionate at about 18.0% (Figure 1D). Notably, the proportion of acetate in the HELP group was significantly lower than in the other three groups, while the LELP group showed a significantly higher proportion of propionate compared to the others (*p* < 0.05) (Figure 1D). The HELP group also had significantly higher proportions of butyric acid compared to the low-energy groups (LEHP and LELP), with the HEHP group also exhibiting significantly higher proportions of butyric acid than the LELP group (*p* < 0.05) (Figure 1D). However, the total VFAs, acetate, and caproate concentrations were similar (*p* > 0.05) across the groups (Figure 1C).

### 3.2 Cecal microbial profiling and differential analysis

**Figure 2:**
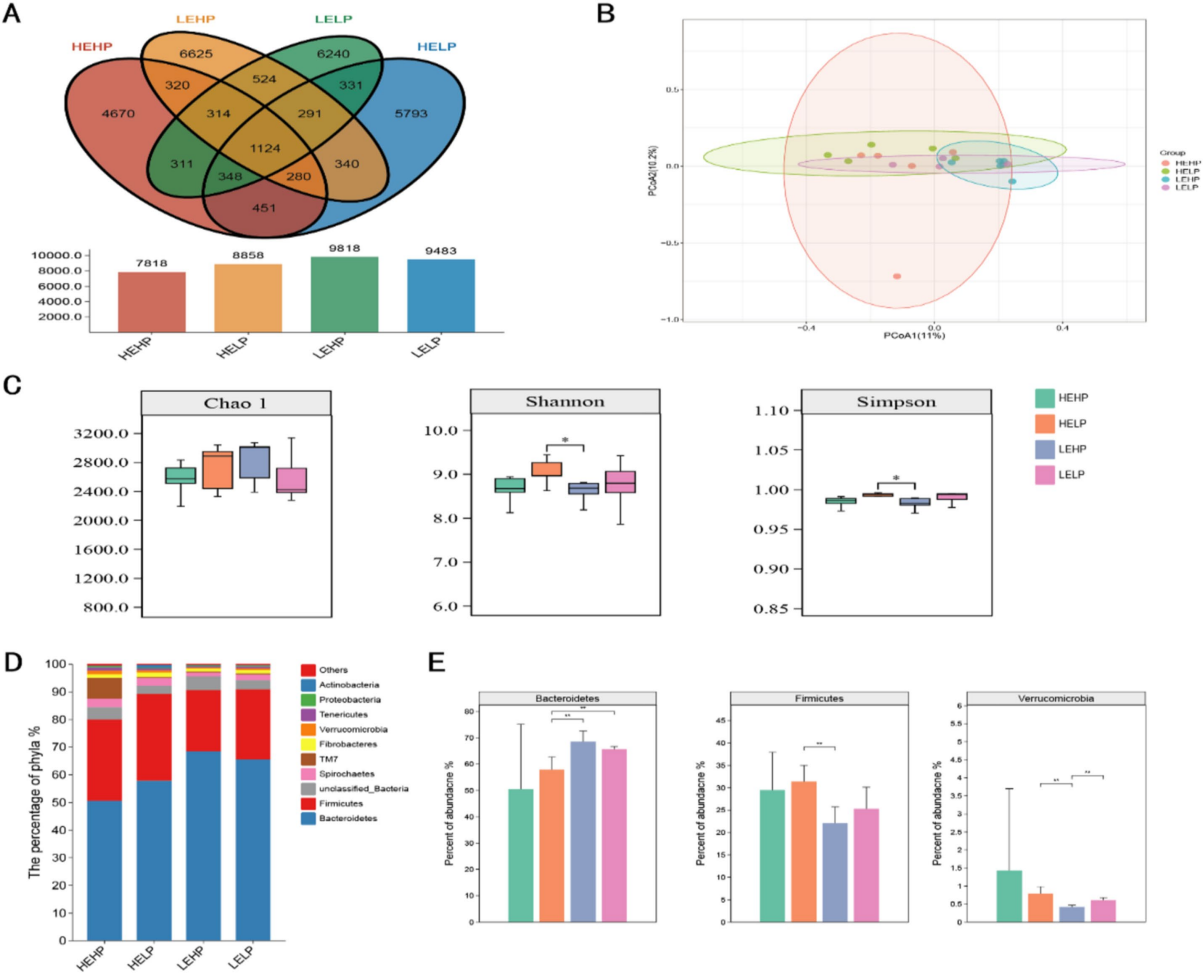
The cecal microbial profile (Venn plot **A**; principal coordinate analysis **B**) and differential analysis (alpha and beta diversities **C**; relative abundance of top 10 phyla **D,E**) of goats fed various energy and protein levels.

**Figure 3:**
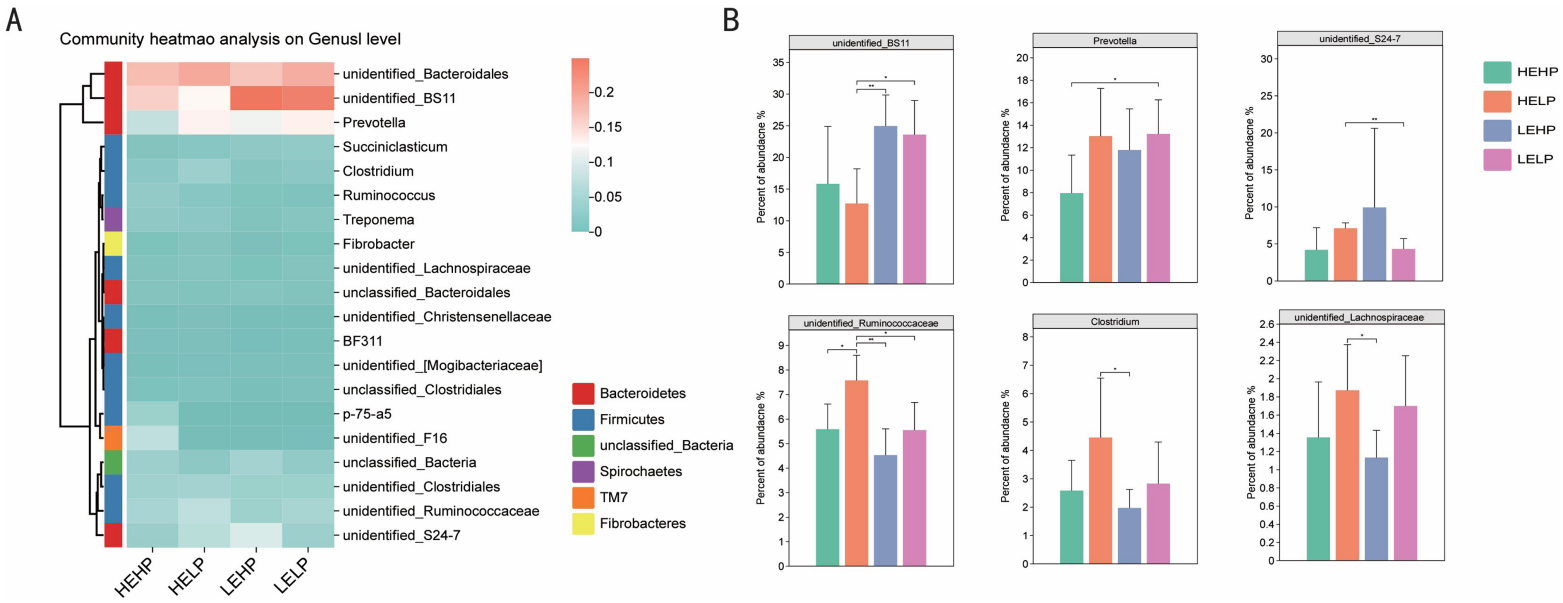
Cecal microbiota composition analysis (heatmap **A**; and relative abundance of top 20 genera **B**) of goats fed different energy and protein levels.

Venn diagram identified the OTUs across four test groups (HEHP, HELP, LEHP, LELP), with counts of 7,818, 8,858, 9,818, and 9,483 OTUs, respectively, totaling 1,124 unique OTUs (Figure 2A). Alpha diversity analysis showed no significant difference in the Chao1 index (species richness) across groups (*p* > 0.05), though Shannon and Simpson indices (diversity) were higher in HEHP than LEHP (*p* < 0.05), indicating increased microbiota diversity in HELP diets. Principal Coordinate Analysis (PCoA) revealed no significant differences in *β* diversity among diets (*p* > 0.05), showing stable community structure (Figures 2B,C). At the phylum level, *Bacteroidetes* and *Firmicutes* were predominant, with LEHP and LELP showing higher *Bacteroidetes* than HELP (*p* < 0.05), and HELP showing higher *Firmicutes* than LEHP (*p* < 0.05) (Figures 2D,E). *Verrucomicrobia* abundance was lower in LEHP compared to HELP and LELP (*p* < 0.05). At the genus level, 311 genera were identified, with key differences in *Clostridium, Prevotella, unidentified_BS11, unidentified_Lachnospiraceae, unidentified_Ruminococcaceae*, and *unidentified_S24-7*. Specifically, *unidentified_BS11* was lower in HELP than low-energy groups, while *unidentified_Ruminococcaceae*, *Clostridium*, and *unidentified_Lachnospiraceae* were higher in HELP compared to LEHP (*p* < 0.05), and *Prevotella* was higher in LELP compared to HEHP (*p* < 0.05) (Figures 3A,B).

### 3.3 Correlation analysis between cecal microbiota and acids

**Figure 4:**
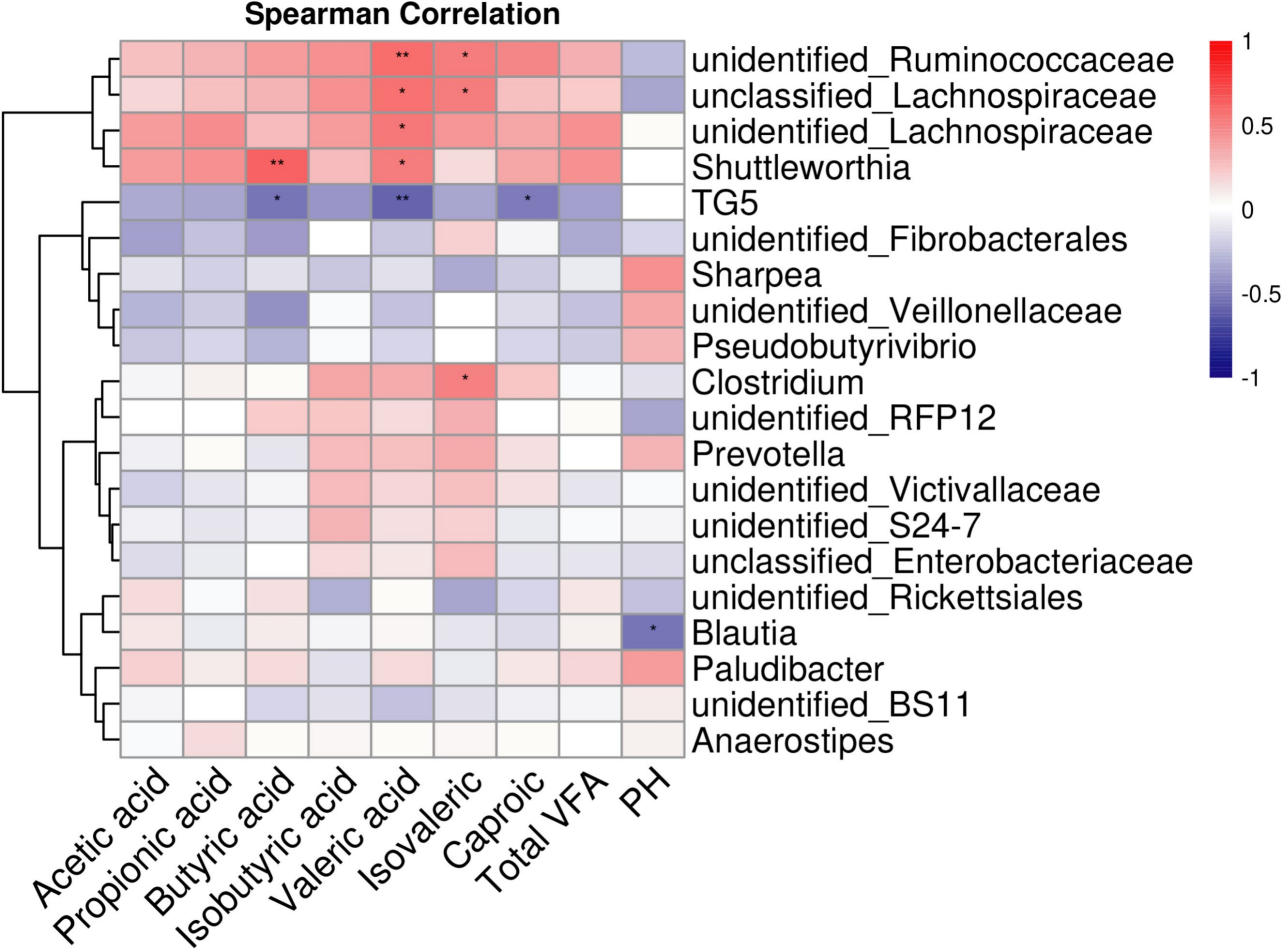
Heat map analysis of cecum fermentation parameters and top-20 microorganisms of genus level of goats fed different dietary energy and protein levels.

Among the 331 identified bacterial genera, the top 20 with significant differences between groups were selected, and the correlation between cecal fermentation parameters and cecal microbiota was analyzed using Spearman’s correlation coefficient (Figure 4). The results showed that butyric acid, valeric acid, isovaleric acid, caproic acid, and pH had significant or extremely significant correlations with cecal microorganisms (*p* < 0.05). In contrast, acetic acid, propionic acid, isobutyric acid, and total VFA did not show significant correlations with cecal microorganisms. The relative abundances of *unidentified_Ruminococcaceae* and *unidentified_Lachnospiraceae* were significantly or extremely significantly positively correlated with valeric acid and isovaleric acid (*p* < 0.05). The relative abundance of *Shuttleworthia* was significantly or extremely significantly positively correlated with butyric acid and valeric acid (*p* < 0.05). Conversely, the relative abundance of TG5 was significantly or extremely significantly negatively correlated with butyric acid, valeric acid, and caproic acid (*p* < 0.05). The relative abundance of *Clostridium* was significantly positively correlated with isovaleric acid (*p* < 0.05), while the relative abundance of *Blautia* was significantly negatively correlated with pH (*p <* 0.05).

### 3.4 VFA absorption, VFA metabolism, relative expression of cytokines, and tight junction protein mRNA in cecum

**Figure 5:**
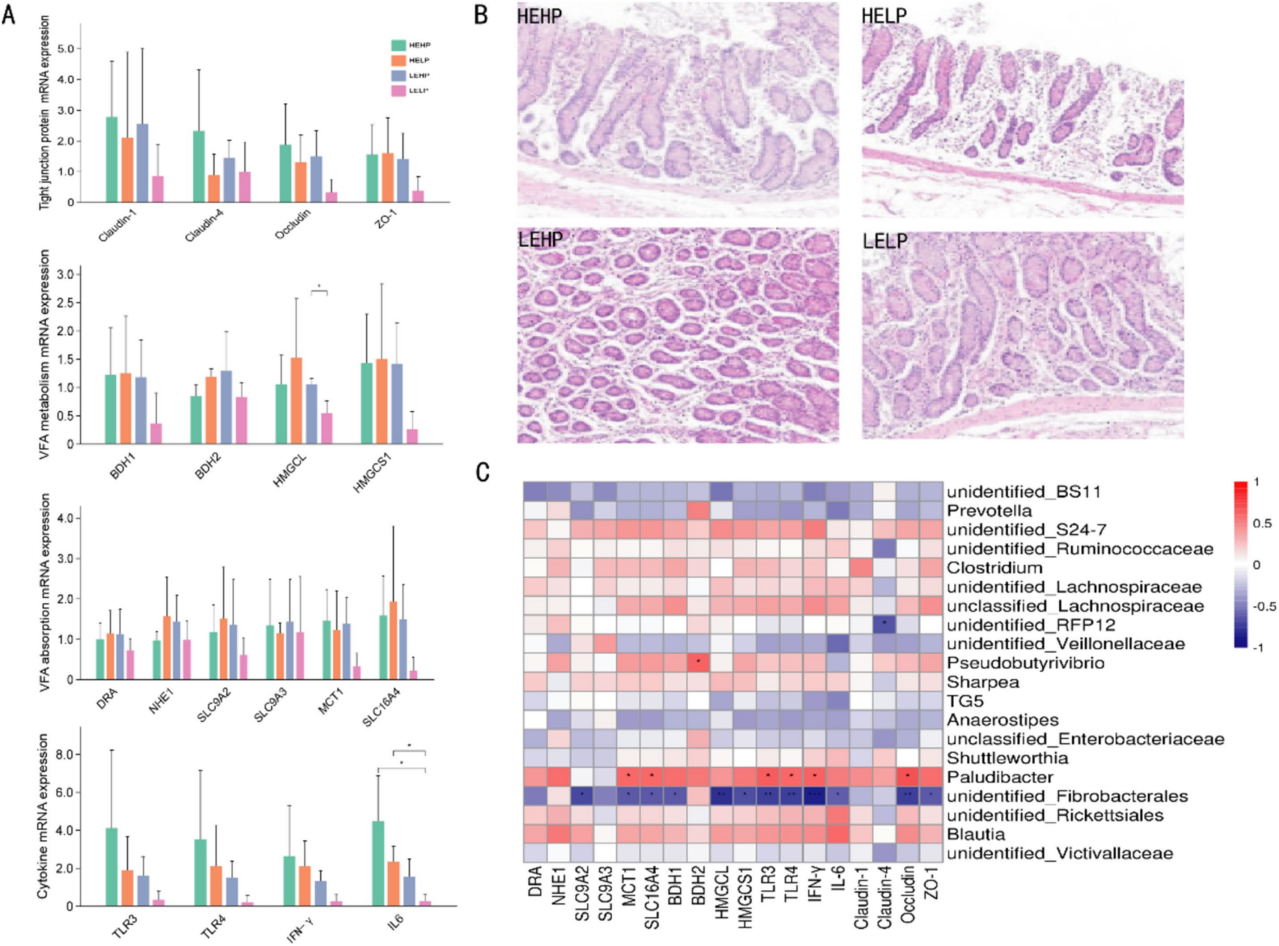
Morphology and related mRNA expression of cecal epithelium. **(A)** mRNA abundances of genes, cytokines, and tight junction proteins associated with VFA uptake and metabolism among the four groups. **(B)** Optical transactions of cecal epithelial tissue of goats fed diets with different energy and protein levels. **(C)** Heat map analysis of mRNA expression of cecal microbiota and associated genes with differences between groups.

Due to the unique physiological structure of the cecum, excessive VFA can induce cecal inflammation. To further investigate this, we collected cecal epithelial tissues to analyze VFA absorption, VFA metabolism, and the relative mRNA expression levels of cytokines and tight junction proteins (Figure 5A). We did not observe significant differences in the mRNA abundances of genes related to VFA absorption or intracellular pH regulation (*p* > 0.05), such as DRA, NHE1, SLC9A2, MCT1, and SLC16A4. Similarly, VFA metabolism in the epithelium showed that genes associated with ketogenesis, such as BDH1, BDH2, and HMGCS1, were not affected by the dietary treatments. However, the mRNA abundance of HMGCL was lower in LELP-fed animals compared to LEHP. Interleukin-6 (IL-6), a pro-inflammatory cytokine, was significantly upregulated in HEHP-fed and HELP-fed animals compared to the LELP after 50 days of feeding (*p* < 0.05). Additionally, we detected the tight junction proteins Claudin-1, Claudin-4, Occludin, and ZO-1, but no significant differences were observed among the four groups (*p* > 0.05). These results suggest that a high-energy (HEHP and HELP) diet may affect the function of the cecal epithelium and induce an inflammatory response. This is supported by light microscopy cross-sections showing sloughing of the cecal epithelial surface in animals fed the high-grain (HG) diet (Figure 5B).

### 3.5 Correlation analysis

The results showed that VFA absorption-related genes, such as MCT-1 and SLC16A4, were positively correlated with the relative abundance of *Paludibacter* (Figure 5C). Additionally, the relative abundance of *Paludibacter* was also positively correlated with cytokines (such as TLR-3, TLR-4, and IFN-*γ*) and the tight junction protein Occludin. Furthermore, our study found that VFA absorption-related genes (such as SLC9A2, MCT-1, and SLC16A4), VFA metabolism-related genes (such as BDH-1, HMGCL, and HMGCS1), cytokines, and tight junction proteins (Occludin and ZO-1) were negatively correlated with the relative abundance of unidentified *Fibrobacterales*. This suggests that the microbial community plays a critical role in host adaptive immunity. These findings indicate that perturbations in the hindgut microbiota can alter the host’s immune system, thereby regulating inflammation.

## 4 Discussion

In this study, changes in the cecal fermentation of goats fed different dietary energy and protein levels were investigated in detail. The pH values in the HEHP and HELP groups were lower than in the LEHP group. Additionally, butyric acid and isobutyric acid concentrations were affected by varying dietary energy and protein levels, with the HELP group showing higher levels compared to the LEHP group. It is well known that feeding increasing levels of dietary energy such as higher inclusion of grain in diets results in the concentrations of total VFA, acetic acid, propionic acid, butyric acid, and lactic acid in the gut of the animals (Zoetendal et al., 1998; Siciliano-Jones and Murphy, 1989) and thus this increasing VFA production poses a greater challenge to maintaining the balance of the gastrointestinal ecosystem (Mertens, 1997). Moreover, a higher accumulation of these organic acids can lead to a decrease in rumen pH and drastic changes in the rumen microbiota, potentially resulting in subacute rumen acidosis (Kleen et al., 2003). A study by DeGregorio et al. (1982) reported that feeding 80% corn grains on a dry basis resulted in cecum engorgement with total VFA contents, along with significantly reduced butyric acid ratios, lactic acid concentrations, acetic acid to propionic acid ratios, and pH. Similarly, another study found increased total VFAs in the cecum when rabbits were transitioned from a low-protein to a high-protein diet (Trocino et al., 2013). In the current study, dietary energy and protein levels did not affect the total VFA content in the cecum of the goats. This may be because the effect of energy levels on the cecum in this study was less pronounced and species differences. Goats have well-developed rumen (primary site of fermentation) and dietary energy substances, such as starch, are primarily fermented into VFAs in the rumen, and any unfermented starch enters the small intestine, where it can be digested and absorbed as glucose, reducing the need for liver gluconeogenesis. Approximately 30% of glucose metabolic requirements can be absorbed from the gut, which helps to increase energy reserves. Our study also revealed that at the same energy level, the proportion of acetic acid increased with the rise in crude protein levels. Our results are consistent with previous research (Manzanilla et al., 2009), which reported that feeding high protein levels increased the cecum acetate of the piglet. Overall, the fermentation parameters of the four groups differed, likely due to variations in the composition of the cecal microbiota suggesting that dietary energy and protein levels influenced the cecum fermentation.

The large and complex microbial ecosystem plays a crucial role in the degradation of feed and the production of end products such as VFAs, microbial proteins, and B vitamins. A study by Zhang et al. (2017) found that a high-grain diet significantly reduced the number of OTUs. The results also indicated that the number of OTUs of rumen epithelial-associated bacteria showed a quadratic significant change over the feeding period with the HG diet. Our findings suggest that feeding the HEHP diet reduced the number of OTUs, whereas giving the LEHP diet increased them. Similarly, at the same protein level, increased energy decreased the number of OTUs. One possible explanation for these changes in the composition of the gastrointestinal bacterial community could be the increase in rapidly fermentable carbohydrates, which can cause a sharp decrease in pH and dynamic changes in the concentrations of butyrate, lactate, and total short-chain fatty acids, as discussed above. The development and nutrient metabolism of the hindgut are closely related to the structure and quantity of the intestinal flora. It has been concluded that feeding patterns, the ratio of dietary concentrate, and the level of dietary nutrients can affect the bacterial diversity and the composition of dominant bacteria in the rumen (Zhang et al., 2020; Liu et al., 2019; Bi et al., 2018). In our study, we performed PCoA analysis on the OTUs and found that the four groups were not highly differentiated. There was no significant difference in the Chao 1 index, but both the Shannon and Simpson indices were significantly higher in the HELP group compared to the LEHP group. This difference in microbial population structure may be due to the increased amount of fermentable substrates in the diet, which are conducive to microbial growth. Additionally, proteins and carbohydrates that are not fully utilized by the small intestine will enter the large intestine and be further fermented and reused by microorganisms. This process results in the production of short-chain fatty acids, which have important physiological functions such as regulating the intestinal flora, enhancing intestinal function, and supplying energy to the host, particularly the intestinal epithelia. In the gastrointestinal tract, it is clear that dietary components play a key role in shaping the prevalence of bacterial phyla and specific intestinal compartments. Dietary factors are indeed key determinants affecting the relative abundance of microorganisms (Petri et al., 2013). The microbial differences between different parts of the gastrointestinal tract may be because when microorganisms flow into the intestine with chyme, the acidic environment can dissolve microbial cells, leading to the degradation or even death of some bacteria. Consequently, the number of rumen microorganisms reaching the hindgut decreases, resulting in changes in the composition of the gastrointestinal microbiota. The cecum is relatively rich in microbial species, with *Firmicutes* and *Bacteroidetes* as the core phyla, accounting for more than 90% of the total intestinal bacteria (O'Hara et al., 2020). *Firmicutes* primarily degrade fibrous substances, while *Bacteroidetes* mainly degrade non-fibrous carbohydrates. The results of this study showed that *Firmicutes* and *Bacteroidetes* were the dominant phyla in the cecum, consistent with other studies using 16S rDNA sequencing (Hook et al., 2011). In this study, the cecal pH of animals in the high-energy diet group was lower, and previous studies have shown that a low-pH gastrointestinal environment is conducive to the growth of *Firmicutes* (Hook et al., 2011), which was also verified in our experiment. Additionally, we found differences in *Verrucomicrobia* at the phylum level. Studies (Sun et al., 2024) have shown that these bacteria may participate in lipid metabolism and are associated with triglycerides and total cholesterol. These results suggest that cecal microbiota are adapting to diets with different energy and protein levels, or they need to become more adaptive over time to changes in the cecal environment caused by feeding with different nutrient levels. Other factors like animal species, physiological stage, and environment may also influence the cecal microflora structure. Moreover, the current study showed that among the top 20 most abundant bacteria, the different genera included *unidentified_BS11*, *Prevotella*, *unidentified_S24-7*, *unidentified_Ruminococcaceae*, *Clostridium*, and *unidentified_Lachnospiraceae* were abundant at the genus level. All these different bacterial genera belong to *Firmicutes* and *Bacteroidetes*. Notably, although the highest relative abundance in the goat cecum was in *Ruminococcus* and *BS_11* (which were not classified), the relative abundance of *Prevotella* was higher in all samples. In healthy animals, *Prevotella* is a dominant microbe in the gastrointestinal tract (Dias et al., 2024), which is consistent with the findings in this study. *Prevotella* can almost complete the degradation of proteins, carbohydrates, and other nutrients by itself, and its distribution in different parts of the gastrointestinal tract is related to its high genetic variation (Stevenson and Weimer, 2007; Mullins et al., 2013). Some bacteria in the family *Ruminococcus* can produce cellulase, amylase, and other carbohydrate-degrading enzymes. *Ruminococcus* helps in the intestinal degradation of cellulose and hemicellulose, producing short-chain fatty acids such as butyric acid and acetic acid, which promote lipid metabolism, produce short-chain fatty acids and lipid products, and facilitate body fat deposition (Niu et al., 2015). Additionally, *Ruminococcus albus* and *Ruminococcus flavefaciens*, both belonging to the *Ruminococcaceae* family, can secrete large amounts of cellulase and hemicellulase, indicating that *Ruminococcaceae* microorganisms have a wide range of carbohydrate degradation activities. In this experiment, the highest relative abundance of microorganisms in the goat cecum was *f_Ruminococcaceae*, which was not classified, and the relative abundance of *g_Ruminococcus* was also high in all samples. Gu et al. (2013) found that *Ruminococcaceae* is the dominant bacterial group in the large intestine and feces of mice, and the results of this experiment are similar to those findings. Our results suggest that there may be active carbohydrate fermentation in the hindgut of goats. Additionally, *Trichomillaceae* were the most abundant phylum in fecal samples of the current study. This phylum can degrade fiber and protein and is associated with improved feed efficiency (Lopes et al., 2019).

Intestinal microbiota can enhance the host’s nutrient absorption and thus promote overall health. In this experiment, the *Ruminococcaceae* family had the highest abundance, and the *Ruminococcus* genus was particularly abundant in cecal contents. *Trichospirillaceae* can digest fibrous substances in the gut to form VFAs, which can reduce gut pH and inhibit harmful microorganisms, thereby maintaining a relatively stable microbial community (Greer et al., 2013). In this study, *unclassified f_Bacteroidaceae, unclassified f_Lachnospiraceae,* and *g_Clostridium* were the dominant bacterial groups in the cecum. Studies have shown that *Bacteroides fragilis*, a member of the Bacteroides genus, can promote the development of the intestinal immune system (Rhee et al., 2004).

It has also been found that *Trichospiraceae* has anti-enteritis properties (Surana and Kasper, 2017). Additionally, several genera within *Lachnospiraceae* are butyricogenic bacteria, capable of producing butyric acid through the fermentation of fiber. Butyric acid can promote the expression of cecal defensin genes, effectively inhibiting the invasion of pathogenic microorganisms (Pryde et al., 2002; Sunkara et al., 2011). *Clostridium* includes *Clostridium butyricum*, a probiotic commonly found in the mammalian intestinal tract. *C. butyricum* can stimulate mucosal immune responses by fermenting fiber and metabolizing butyric acid, thereby inhibiting the growth of harmful intestinal bacteria (Mo et al., 2015). These results suggest that cecal microbes play a crucial role in maintaining gut health. Furthermore, all three of these microorganisms secrete cellulase, which aids the host in digesting cellulose (Bian et al., 2013; Zeng et al., 2015; Daly et al., 2012; Zhu et al., 2011). Additionally, our study found that the TG5 genus, belonging to *Dethiosulfovibrionaceae*, was negatively correlated with VFA content. Previous studies have indicated that an increased abundance of *Dethiosulfovibrionaceae* can serve as a potential biomarker for myocardial fibrosis (Marcinkiewicz et al., 2017). Moreover, the growth of *Desulfovibrio* is inhibited in low-pH environments (Huang et al., 2019). The results of this study once again suggest that there may be active carbohydrate fermentation in the cecum of goats. Previous research has shown that the hindgut of ruminants can compensate for the fibrous material not fully digested in the rumen (Wang et al., 2017; Jiao et al., 2014). This suggests that cecal microorganisms may regulate intestinal immune function primarily by affecting digestive metabolites.

The gastrointestinal tract of ruminants hosts a rich variety of microbial flora, with different regions emphasizing distinct microbial functions. For example, rumen microorganisms primarily focus on digesting and degrading nutrients, especially fibrous materials, while intestinal microorganisms play a crucial role in maintaining the host’s intestinal health, in addition to compensating for and digesting nutrients not fully broken down in the rumen (Dodd et al., 2017; Kadoki et al., 2017; Cervantes-Barragan et al., 2017; Koppel et al., 2017; Bauer et al., 2018). Furthermore, digestive tract microorganisms are closely linked to local and systemic immune responses, preventing the invasion of pathogenic bacteria and maintaining intestinal homeostasis (Kataoka, 2016). Previous studies have also shown that mice fed a high-protein diet can experience increased severity of inflammatory bowel disease (Ng and Ananthakrishnan, 2019), and a high-fat diet can promote neuroinflammation (Kim et al., 2019). In this study, we observed changes in VFA content and microbial composition in the cecum when feeding diets with different energy and protein levels. We found that the tight junctions between cecal epithelial tissues in the experimental group showed erosion and cell necrosis. Previous research has indicated that high levels of hindgut fermentation can lead to inflammation and laminitis, which may be induced by amines, endotoxins, or bacteria capable of penetrating the intestinal barrier (Gozho et al., 2006; Crawford et al., 2007). In this study, we did not find any significant differences in the mRNA abundance of genes related to VFA absorption among the four groups. However, HMGCL, a gene related to VFA metabolism, was down-regulated in the LELP group, which may be related to the change in cecal pH. Additionally, we measured the mRNA expression of genes related to cytokines and inflammatory factors. The mRNA expression of the cytokine IL-6 in the LELP group was significantly down-regulated compared to the HEHP and HELP groups, suggesting that immune system activation may be involved in the adaptation of the cecal epithelium to a high-nutrient diet. IL-6 is a multifunctional, pleiotropic cytokine involved in the regulation of immune response, acute phase response, and inflammation (Chang et al., 2014). Generally, the occurrence of inflammation affects the mRNA expression of genes related to tight junction proteins, but no such phenomenon was observed in this study. We speculate that this might be due to the adaptation of microorganisms to changes in nutrient levels, with the carbon metabolism of the cecal microbiome being altered by the introduction of a high-energy, high-protein diet, and adapting over time. Previous studies have reported that ruminants can adapt to a high-grain diet, which requires at least 3 weeks of rumen microbial adaptation (Tajima et al., 2001) and rumen epithelial morphological and functional adaptation (Górka et al., 2017). A highly interdependent relationship exists between the microbes in the gut microbiota system and the host, and the diversity of gut microbes has been proposed as a new marker to assess gut health and metabolic capacity (Clarke et al., 2014). The interaction between intestinal symbionts and the immune system plays an active role in maintaining overall health by promoting the growth of immune cells, shaping immune responses, and maintaining the integrity of the intestinal barrier (Zhou et al., 2024; Rinninella et al., 2019). To investigate the correlation between gene expression and cecal microbial abundance, we conducted a heatmap analysis. The results showed that *Pseudobutyrivibrio* belongs to the *Trichospirillum* genus, which is part of the *Trichospirillaceae* family. This family is a major producer of short-chain fatty acids and is known to enhance epithelial integrity, protect the barrier, and inhibit inflammation (Sun et al., 2021; Sun et al., 2021). Additionally, our study found that *Paludibacter* was positively correlated with the expression of genes related to VFA absorption, as well as genes associated with inflammatory factors and tight junction proteins. Previous studies have shown that *Paludibacter* is significantly correlated with feed utilization efficiency (Myer et al., 2017), and can help the host capture more protein and energy materials (Huang et al., 2022; Zhang et al., 2020). Furthermore, in our study, *unidentified_Fibrobacterales* was associated with RPF12 and had an effect on the expression of VFA-related genes in the cecum and intestinal health. Specifically, *unidentified_Fibrobacterales* were correlated to varying degrees with VFA absorption and transport genes, cytokines, and tight junction proteins. This indicates a long-term interdependence and restriction relationship between the microorganisms in the cecum and the host, as well as between different microorganisms. The dynamic relationship among these microorganisms is closely related to the absorption and transport of nutrients in the body. The intestinal microbiota also provides other beneficial functions to the host, such as balancing immune function, stabilizing the intestinal environment, and improving the health level of animals (Shapira, 2016; Hasegawa and Inohara, 2014).

## 5 Conclusion

Based on the results, feeding different energy and protein levels altered the cecal microbiota composition. The dominant phyla were *Bacteroidetes* and *Firmicutes* across the groups. At the genus level, 311 genera were identified, with *Clostridium*, *Prevotella*, *unidentified_BS11*, and others showing significant variation. Additionally, the analysis of VFA metabolism, absorption, cytokine expression, and tight junction protein mRNA in cecal tissue revealed that MCT-1 and SLC16A4 genes are linked to VFA absorption, positively correlated with *Paludibacter*, which was associated with immune markers (TLR-3, TLR-4, IFN-*γ*) and Occludin expression. In contrast, VFA-related genes and tight junction proteins negatively correlated with *unidentified Fibrobacterales*, suggesting a microbial role in adaptive immunity. The results of this study provide theoretical guidance for the practical application of diets with varying energy and protein levels in fattening goats.

## Data Availability

The data presented in the study are deposited in the NCBI BioProject database with accession number PRJNA1214186. The data can be assessed by using this link: https://www.ncbi.nlm.nih.gov/bioproject/1214186.
